# Clinical Profile and Treatment Outcome in MOGAD: A Single-Center Case-Series Study in Guiyang, China

**DOI:** 10.3389/fneur.2022.830488

**Published:** 2022-04-06

**Authors:** Xiaoyang Lei, Shipeng Guo, Shengnan Cui, Yin Pu, Anni Zhang, Dian He

**Affiliations:** Department of Neurology, The Affiliated Hospital of Guizhou Medical University, Guiyang, China

**Keywords:** MOGAD, case-series, CLIPPERS, area postrema syndrome, prognosis

## Abstract

**Background:**

The clinical spectrum of myelin oligodendrocyte glycoprotein antibody-associated disease (MOGAD) is expanding over time. However, the long-term management and prognosis of this disorder are still controversial. Therefore, this study aimed to report the clinical profiles and treatment outcomes of MOGAD in our center.

**Methods:**

This was a single-center case-series study. Clinical and para-clinical data, along with treatment outcomes of patients with MOGAD were analyzed.

**Results:**

A total of 27 patients were identified, of which 19 (70%) patients were women, and the median age at disease onset was 40 years (range 20–67). A total of 47 episodes were observed, with optic neuritis (53%) being the most frequent presentation and 60% of them were unilateral. Other presentations included rhombencephalitis (RE) (17%), limbic encephalitis (9%), simultaneous optic neuritis and myelitis (9%), acute disseminated encephalomyelitis (ADEM)-like presentation (6%), myelitis (4%), and ADEM (2%). One patient presenting with RE also met the diagnostic criteria of area postrema syndrome (APS). Another patient with RE presented with imaging characteristics of chronic lymphocytic inflammation with pontine perivascular enhancement responsive to steroids (CLIPPERS). A total of 29 lumbar punctures were recorded, among which an elevated protein level was found in 34% of the samples, pleocytosis was found in 14% of the samples, and positive intrathecal oligoclonal bands were found in 19% of the patients. One patient was found to have anti-N-methyl-D-aspartate receptor antibodies both in his serum and cerebrospinal fluid. Intravenous methylprednisolone (IVMP) was administrated for 85% of the attacks while both IVMP and intravenous immunoglobulin were for 6% of the attacks. Moreover, nine patients received maintenance therapy. Among them, six patients were treated with mycophenolate mofetil, three patients were treated with prednisone, rituximab, and teriflunomide, respectively. The median follow-up period was 20 months (range 6–127). At follow-up, twelve (44%) patients experienced a relapsing course, and the median time to the first relapse was 9.5 months (range 2–120). The median Expanded Disability Status Scale score at nadir was 3.5 (range 2–8) and was 0 (range 0–3) at the last follow-up.

**Conclusion:**

The clinical spectrum of MOGAD is heterogenous, wherein APS and CLIPPERS-form can occur. The long-term outcome of MOGAD seems benign. Further studies are warranted to determine the risk factors of relapse and identify the optimal steroid-sparing agents.

## Introduction

Myelin oligodendrocyte glycoprotein antibody-associated disease (MOGAD) is an inflammation disorder of the central nervous systems (CNS), which has been considered as a distinct CNS demyelinating disease that differs from multiple sclerosis (MS) and neuromyelitis optica spectrum disorder (NMOSD) in many aspects (1). With an increasing number of studies, the known clinical features of MOGAD are no longer limited to optic neuritis (ON), myelitis (MY), and acute disseminated encephalomyelitis (ADEM). This is because other unexpected manifestations can also occur, such as area postrema syndrome (APS), the imaging characteristics of chronic lymphocytic inflammation with pontine perivascular enhancement responsive to steroids (CLIPPERS), meningoencephalitis, ADEM-like form presentations, leukodystrophy, and peripheral nervous system involvement (2–6). Therefore, it is important to recognize these new features for the diagnosis of MOGAD. The treatment strategies for MOGAD are mainly adopted from NMOSD and MS, but experts suggest not all patients should receive long-term immunotherapy because of the features of the disease course and the uncertainty of relapse (1). Moreover, immunotherapies for MOGAD do not always show its effectiveness as it has been for other diseases (7). Although the long-term outcomes of MOGAD seem benign, relapses still occur (1). So far, there are still many unknowns regarding the clinical features and the prognosis of MOGAD. In this study, we aimed to report the clinical findings and treatment outcomes of MOGAD in our center.

## Methods

This was a single-center, case-series study, and the collection of patients was consecutive. This study was approved by the Institutional Review Board of the Affiliated Hospital of Guizhou Medical University, and all included patients provided written informed consent.

The myelin oligodendrocyte glycoprotein (MOG)-immunoglobulin G (MOG-IgG) test was performed using live cell-based assays, which employed human embryonic kidney cells expressing full-length human MOG incubated with the patient's serum and secondary anti-human IgG1 antibody.

All included patients fulfilled the following criteria: (1) visited our hospital between April 2018 and July 2021; (2) aged >18 years old; (3) met the 2018 MOGAD diagnostic criteria (8); (4) had sufficient clinical data available; (5) had a follow-up period of at least 6 months.

Demographic and clinical data, results of serological and cerebrospinal fluid (CSF) analysis, MRI findings, details of acute treatment, and maintenance treatment were collected from the clinical records. Neurological involvements were categorized into ON, MY, simultaneous ON and MY, rhombencephalitis (RE), limbic encephalitis (LE), ADEM, and ADEM-like form. MRI data were acquired using a 3T-MRI (Siemens, Germany) scanner, consisting of the following sequences: T1, T2, flair, and T1 contrast imaging of the brain, spinal cord, and optic nerves. Most patients received therapy with intravenous methylprednisolone (IVMP) during the acute period, with some of them receiving treatment with intravenous immunoglobulin (IVIG) which depended on the severity of the disease and the patients' will. Maintenance treatments were given depending on several conditions, such as the severity of the disease, the patient's affordability, and the frequency of a relapse.

A relapse was defined as any new CNS symptom/sign lasting more than 24 h in the absence of other causes. The Expanded Disability Status Scale (EDSS) was used to evaluate the therapeutic effects. Patients were followed up through outpatient consultation and telephone inquiries every 2 or 3 months.

### Statistical Analysis

Categorical variables were presented as frequencies and percentages, and the continuous variables are presented as the median and range. Analyses were conducted using SPSS, version 26 (Chicago, IL, USA).

## Results

### Demographic Data and Clinical Features

A total of 28 patients fulfilling the 2018 MOGAD diagnostic criteria were identified in our center. One patient was excluded because of insufficient clinical data, whereas 27 patients' data were analyzed (Table 1). A total of 19 (70%) patients were women, the median age at disease onset was 40 years (range 20–67).

**Table 1:** Investigation findings of the cohort.

**Demographics**	**Value**
Patients, *n*	27
Female, *n* (%)	19 (70%)
Median age of onset (range),y	40 (20–67)
**Clinical features**, ***n*** **(%), 47 attacks**	
ON	25 (53%)
Unilateral ON	15 (60%)
RE	8 (17%)
LE	4 (9%)
Simultaneous ON and MY	4 (9%)
ADEM-like	3 (6%)
MY	2 (4%)
ADEM	1 (2%)
**CSF finding**	
Elevated protein, *n* (%)	10/29 (34%)
Pleocytosis, *n* (%)	4/29 (14%)
Oligoclonal bands, *n* (%)	3/19 (16%)
**Other autoimmune disease**, ***n*** **(%)**	
Hashimoto thyroiditis	1 (4%)
Other autoantibodies, *n* (%)	7 (26%)
CCP	4 (57%)
ASO	1 (14%)
Anti-Sm	1 (14%)
Anti-NMDAR	1 (14%)
**Acute therapy**, ***n*** **(%)**	
IVMP	40/47 (85%)
IVMP + IVIG	3/47 (6%)
**Attack prevention treatment**, ***n*** **(%)**	
Prednisone	1/9 (11%)
MMF	6/9 (67%)
RTX	1/9 (11%)
Teriflunomide	1/9 (%)
**Prognosis**	
Median disease duration (range), m	20 (6–127)
Relapse, *n* (%)	12 (44%)
Median time until first relapse (range), m	9.5 (2–120)
Median EDSS at nadir, range	3.5 (2–8)
Median EDSS at last follow-up, range	0 (0–2)

*ON, optic neuritis; RE, rhombencephalitis; LE, limbic encephalitis; MY, myelitis; ADEM, Acute disseminated encephalomyelitis; CSF, cerebrospinal fluid; CCP, cyclic citrullinated peptide antibody; ASO, antistreptolysin O; Sm, Smith antibody; NMDAR, N-methyl-D-aspartate receptor; IVMP, intravenous methylprednisolone; IVIG, intravenous immunoglobulin; MMF, mycophenolate mofetil; RTX, rituximab; EDSS, Expanded Disability Status Scale*.

**Figure 1:**
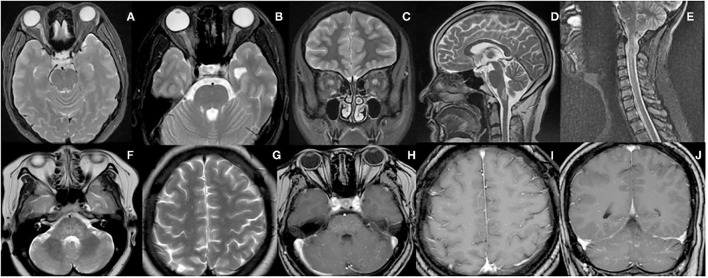
MRI features of the representative patients. **(A)** A 40-year-old woman presented with acute vision loss of the right eye, and MRI scans revealed hyperintense lesion in the right optic nerve on T2-weighted images. **(B,C)** A 26-year-old woman presented with acute vision loss of both eyes, and MRI scans revealed hyperintense lesions in the bilateral optic nerves on axial and coronal T2-weighted images. **(D)** A 45-year-old woman presented with persistent nausea and vomiting, and MRI scans revealed a hyperintense lesion in the medulla on sagittal T2-weighted images. **(E)** A 44-year-old woman presented with numbness in her right upper extremity, and a hyperintense lesion in the cervical spinal cord was shown on sagittal T2-weighted images. **(F–J)** In the CLIPPERS-form case, extensive lesions were shown in the pons, pontibrachium, cerebellum, and hemispheres on T2-weighted images and after gadolinium-enhanced T1-weighted images, and a typical peppering sign was seen in both supratentorial and infratentorial [T2 scans are F&G while T1 gadolinium contrast scans are (**H–J**)].

After a median disease duration of 20 months (range 6–127), a total of 47 CNS demyelinating episodes were observed. Among them, 25 (53%) episodes were ON, of which 15 (60%) were unilateral. Other attack forms according to the occurrence rate were as follows: RE (*n* = 8, 17%), LE (*n* = 4, 9%), simultaneous ON and MY (*n* = 4, 9%), ADEM-like form (*n* = 3, 6%), MY (*n* = 2, 4%), and ADEM (*n* = 1, 2%). Furthermore, twelve (44%) patients had a relapsing course with a total of 20 relapsing episodes. Among them, ON was still the most common presentation (50%), and 70% of them were unilateral. In the rest of the relapsing episodes (*n* = 10), RE, LE, and MY accounted for 25, 20, and 5%, respectively. It is worth mentioning that 1 patient presenting with RE also met the diagnosis of APS (9). This patient initially had persistent nausea and vomiting, subsequently developed dysphagia, numbness in her right face and left limb, with an obvious lesion located in the dorsal medulla on brain MRI (Figure 1). Another patient presenting with RE was once diagnosed with CLIPPERS, who was described in more detail below.

### CSF and Serological Analysis

A total of 29 lumbar punctures were recorded, of which an elevated protein level was found in 10 (34%) of the samples, while pleocytosis was found in 4 (14%) of the samples. A total of 19 patients were tested for intrathecal oligoclonal bands but only 3 (19%) were positive. All patients were evaluated for serum antibodies. Among them, one patient was seropositive for anti-thyroid peroxidase autoantibody, and a diagnosis of Hashimoto's thyroiditis was subsequently made. Furthermore, four patients were seropositive for anti-cyclic citrullinated peptide antibodies. Serum anti-streptolysin O was found in 1 patient, and another patient tested seropositive for anti-Smith antibody. However, none of these 6 patients met the diagnostic criteria of connective tissue disorders. One young man was found to have anti-N-methyl-D-aspartate receptor antibody (anti-NMDAR) in his serum and CSF.

### Treatment

Regarding the treatments for the 47 attacks, IVMP was administrated for 40 (85%) attacks. Combination therapy of IVMP with IVIG was given to 3 (6%) attacks because of severe encephalopathy, and treatment was not given to the rest of the 4 attacks. In the remission stage, six (67%) patients were treated with mycophenolate mofetil (MMF), and the rest of 3 patients received monotherapy with prednisone, rituximab, and teriflunomide, respectively.

### Follow-Up

The median follow-up period was 20 months (range 6–127), and one patient has been considered seronegative NMOSD for many years until the technology of the MOG-IgG test was available in our center. The median time to the first relapse was 9.5 months (range 2–120), wherein four (33%) patients experienced a relapse within 6 months after onset and seven (58%) patients relapsed within 1 year. The recurrent events of 4 patients were related to prednisone tapering or withdrawal. The median EDSS score was 3.5 (range 2–8) at nadir and was 0 (range 0–3) at the last follow-up.

### A Special Case: Mimicking CLIPPERS

A 23-year-old woman with unremarkable medical history was referred to a local hospital with 10 days of dizziness, nausea, and vomiting. Neurological examination revealed horizontal nystagmus and bilateral ankle clonus. The blood tests demonstrated hypochromic microcytic anemia, while all other routine tests were normal. The results of the CSF analysis were within normal limits, with no evidence of oligoclonal bands. Brain MRI revealed multiple patchy and nodular hyperintensities in pontocerebellar areas and posterior horn of lateral ventricle on T2-weighted images. Contrast-enhanced MRI scan showed punctate and curvilinear lesions (images not shown). According to the clinical manifestations of RE and the morphology of lesions on MRI (10), a diagnosis of CLIPPERS was suspected. Subsequently, IVMP at a dose of 1 g/day was administrated for 3 days, followed by oral prednisone at a dose of 60 mg/day. The symptoms were completely resolved shortly. Nevertheless, MOG-IgG was seropositive (1:100). The patient was diagnosed with MOGAD and discharged with slow tapering of prednisone by 5 mg every 2 weeks.

The patient experienced 3 relapses during the 1-year follow-up. The first relapse occurred when the daily dose of prednisone was tapered to 40 mg. The relapsing symptoms were similar to those at the onset. The patient was re-treated with IVMP followed by oral prednisone on the same regimen and responded well. When the daily dose of prednisone was tapered to 20 mg, she relapsed with gait ataxia, dysarthria, and ophthalmoplegia, and these symptoms remained sensitive to IVMP and prednisone. MMF 1 g/day was added at discharge to prevent further relapse.

The third relapse occurred with similar symptoms of the previous attack when the daily dose of prednisone was tapered to 20 mg. Subsequently, the patient was referred to our hospital. Long-term steroid therapy caused severe side effects, such as weight gain and cushingoid appearance. Serum MOG-IgG re-tested positive (1:100) 7 months later after the first test. Brain MRI revealed more extensive lesions with involvements of both the midbrain and hemispheres. Contrast-enhanced MRI scans showed these lesions with a typical “peppering sign” (Figure 1). Finally, a definite diagnosis of MOGAD was made. Considering the features of lesions on MRI were consistent with the characteristics of CLIPPERS (10), we thought there was a CLIPPERS-like form of MOGAD. Unlike the previous relapses, the patient did not respond well to IVMP. Therefore, a 5-day therapy with IVIG at a dose of 0.4 g/kg/day was added, and the symptoms gradually subsided. Considering the side effects of steroids, the patient was discharged with relatively rapid tapering of prednisone by 5 mg every 10 days. Meanwhile, the dose of MMF was increased to 1.5 g/day to prevent further relapse.

## Discussion

We analyzed the clinical data of 27 cases with MOGAD in our center, and a predominance of women at 70% was found. Previous reports indicated no major sex differences in such patients (11). The cause of this inconsistency in gender distribution may be due to the relatively small number of cases included in our center.

Optic neuritis was the most common clinical manifestation at onset and relapse, and more than half of them were unilateral. These findings were consistent with the previous studies (1, 12). A total of 8 attacks of RE in 3 cases were recorded. Among them, one patient presented with APS, which is a rare manifestation of MOGAD. In a Korean cohort (13), 2/107 (1.9%) adults with MOGAD experienced APS. On the other 3 cohorts from Europe and America, it was revealed that APS was presented in 1/50 (2%), 3/75 (4%), 1/173 (0.6%) patients, respectively (12, 14, 15). However, the occurrence rate of APS is far less than that in NMOSD (13). Thus, APS as a phenotype of MOGAD should be noticed more often. MOG-IgG should be tested in patients presenting with APS because MOGAD differs from NMOSD in terms of treatment and prognosis (16). As described separately in detail above, another patient with RE presented with a CLIPPERS-like phenotype. This phenotype is also extremely rare in MOGAD. To the best of our knowledge, MOG-IgG has been detected in only 5 patients with CLIPPERS so far (3, 17, 18). The lesions of these 5 patients were confined to the hindbrain. However, it was exceptional that our patient presented with a typical “peppering sign” in both the hindbrain and hemispheres. Obeidat et al. (18) proposed 3 possibilities for these cases: 1) this is an overlap syndrome of the two conditions, 2) this is a CLIPPERS-like phenotype of MOGAD, and 3) CLIPPERS can be a syndrome of several conditions. We prefer the latter two viewpoints based on the evidence that several cases with CLIPPERS were observed to convert to other diseases after initial diagnosis (19–21). Furthermore, there is no biomarker for the diagnosis of CLIPPERS, and the clinical diagnosis highly depends on the imaging characteristics of MRI and treatment response to corticosteroids. These situations bring great uncertainty to a definite diagnosis of CLIPPERS in the absence of pathological evidence. The last remarkable patient was a 22-year-old man who presented with ADEM at the onset. It has been reported that ADEM is an age-dependent phenotype in MOGAD, in which only ~5% of adults presented (1). In addition, ADEM-like phenotype was observed in 3 patients with MOGAD, while this particular phenotype is distinct from NMOSD and MS. After all, the latter two conditions have their own inherent and recognizable features.

The CSF analyses in our study showed pleocytosis and slightly elevated protein levels in 14% and 34% of patients, respectively, which are much lower than those in previous studies (12, 22). In addition, CSF oligoclonal bands were found in 16% (3/19) of patients, and this data is consistent with the previous studies (12, 22).

It is reported that coexistent autoimmune diseases and autoantibodies were more common in NMOSD in comparison with MOGAD (23). None of the patients included in the current study had connective tissue diseases. Only 1 patient was diagnosed with Hashimoto thyroiditis, but no association of MOGAD with Hashimoto thyroiditis was found. Several studies have reported that MOG-IgG coexisted with NMDAR-Ab and other neuronal antibodies (e.g., GABA-AR and CASPR2) (24). The occurrence rate of MOG-IgG coexisted with NMDAR-Ab is much higher than that of AQP4-IgG. In 2 Chinese cohorts, NMDAR-Ab was found in 5/87 (6%) and 5/42 (12%) of patients with MOGAD (4, 25). Such a kind of patient often presented with encephalitic manifestations, but ON could also be presented (26). In the current study, we also observed a 24-year-old young man carrying serum NMDAR-Ab who presented with recurrent encephalitis and had ON during his first attack.

More than half of the patients had an EDSS score of 0 at the last follow-up. Among them, three patients had the highest EDSS score of 3, and they all had a relapsing course but did not receive treatment during one of the relapses. Untreated attacks left them with residual disabilities, however, a full recovery to baseline was achieved after timely treatment during the subsequent relapses. These findings indicate a favorable long-term outcome of MOGAD provided that timely and full therapies were given for each attack, which supports the results from other studies. In a multi-center cohort of 61 patients from France (27), the median EDSS score was 1 (range 0–7.5) after a median follow-up period of 177 months (range 98–657). In another study including 29 patients from Mayo Clinic (28), the median EDSS score was 2 (range 0–10) after a median follow-up period of 14 years (range 9–31). No definite factors associated with poor outcomes were found in these studies. However, two patients in the cohort of Mayo Clinic had a final EDSS score of 7 and 10, respectively, because of cumulative disability resulting from a high frequency of relapse (29). In general, these data suggest untreated attacks were the primary reason for worse clinical outcomes in MOGAD.

As is well-known, long-term immunotherapy should be recommended for the prevention of attacks in MS and NMOSD as soon as the diagnosis is made. In contrast, immunotherapy is currently not a reasonable option for all patients with MOGAD after the initial attack since some of such patients have a monophasic course. It is more reasonable to provide immunotherapy for relapsing patients (1). However, it seems that the frequency of recurrence is related to the length of the disease course. As with a recurrence of 44% in our study with a median short duration of 20 months, such a low recurrence rate (28–59%) was reported in cohorts with a shorter period of follow-up (median time 15–42 months) (5, 30–32). By contrast, studies with long-term follow-up (75 months to 14 years) showed most patients (80–95%) had relapses eventually (27, 28, 33). One patient in the current study had a relapse 12 years later after onset, and then MMF was added to prevent further attack. These findings suggest that patients with MOGAD may relapse over time. This phenomenon also raises another question regarding whether it is worthwhile to receive long-term immunotherapy to prevent a relapse after many years since the initial attack. Furthermore, the attendant risks of long-term immunotherapy and the financial burden should be considered. More importantly, the key point is to determine which factors can predict a relapse.

Consistent with the results from other reports (1), a recent study attempted to identify potential contributors for relapse, but none of the included factors was related to relapse, including age, gender, race, clinical phenotype, antibody status, and CSF results (31). A study aimed to find ethnic differences between patients with MOGAD from Japan and Germany (34), and the results showed that German patients had a high frequency of a relapsing course (75 vs. 36.4%). In addition, the results from several studies suggested that the persistent existence of MOG-IgG was a predictor for relapse, and immunotherapy should be considered in such patients even without a second attack (5, 8, 35, 36). However, in the same cohort, several patients with a negative conversion of MOG-IgG still suffered a second attack, while persistent MOG-IgG was tested in patients with a monophasic course (36). These paradoxical results suggest the status of the antibody may not be a qualified candidate. In line with our cohort, other studies also suggested that some patients were steroid-dependent (37), and relapse often occurred when prednisone was tapered to a low dose, soon after cessation, or during a rapid tapering. Therefore, experts have suggested a slow tapering and prolonged duration of steroid therapy (37, 38). However, this may result in severe adverse effects, so optimal steroid-sparing agents are still needed.

Our study had several limitations. The inherent bias in the retrospective study was the main limitation. Also, the small samples size and relatively short follow-up period might not have fully reflected the overall characteristics of MOGAD. However, in the absence of large-size, multi-center studies, the current study expanded the clinical features and added data to long-term outcomes of MOGAD.

## Conclusion

In conclusion, the clinical characteristics of MOGAD are heterogeneous, in which APS and CLIPPERS-form can occur. The long-term prognosis is relatively favorable in most patients. However, it is notable that some patients may have highly-relapsing courses and accumulating disabilities. Large-size, multi-center studies are warranted to determine the risks of relapse.

## Data Availability Statement

The original contributions presented in the study are included in the article/supplementary material, further inquiries can be directed to the corresponding authors.

## Ethics Statement

The studies involving human participants were reviewed and approved by the Institutional Review Board of the Affiliated Hospital of Guizhou Medical University. The patients/participants provided their written informed consent to participate in this study.

## Author Contributions

XL designed and drafted the manuscript. SC and YP collected the data from medical records. SG and AZ contacted the patients and followed up regularly. DH designed and supervised the study. All authors contributed to the article and approved the submitted version.

## Funding

This research was supported by the National Natural Science Foundation of China (Grant No. 82060235).

## Conflict of Interest

The authors declare that the research was conducted in the absence of any commercial or financial relationships that could be construed as a potential conflict of interest.

## Publisher's Note

All claims expressed in this article are solely those of the authors and do not necessarily represent those of their affiliated organizations, or those of the publisher, the editors and the reviewers. Any product that may be evaluated in this article, or claim that may be made by its manufacturer, is not guaranteed or endorsed by the publisher.
